# Differential expression patterns of housekeeping genes increase diagnostic and prognostic value in lung cancer

**DOI:** 10.7717/peerj.4719

**Published:** 2018-05-09

**Authors:** Yu-Chun Chang, Yan Ding, Lingsheng Dong, Lang-Jing Zhu, Roderick V. Jensen, Li-Li Hsiao

**Affiliations:** 1 Division of Renal Medicine, Brigham and Women’s Hospital, Harvard Medical School, Boston, MA, United States of America; 2 Research Computing, Harvard Medical School, Boston, MA, United States of America; 3 Department of Nephrology, The Eighth Affiliated Hospital, Sun Yat-sen University, Shenzhen, China; 4 Department of Biological Sciences, Virginia Polytechnic Institute and State University (Virginia Tech), Blacksburg, United States of America

**Keywords:** Housekeeping genes, Expression patterns, Lung adenocarcinoma, Small cellcarcinoma, Squamous cell carcinoma, Non-small cell carcinoma, Diagnosis and prognosis

## Abstract

**Background:**

Using DNA microarrays, we previously identified 451 genes expressed in 19 different human tissues. Although ubiquitously expressed, the variable expression patterns of these “housekeeping genes” (HKGs) could separate one normal human tissue type from another. Current focus on identifying “specific disease markers” is problematic as single gene expression in a given sample represents the specific cellular states of the sample at the time of collection. In this study, we examine the diagnostic and prognostic potential of the variable expressions of HKGs in lung cancers.

**Methods:**

Microarray and RNA-seq data for normal lungs, lung adenocarcinomas (AD), squamous cell carcinomas of the lung (SQCLC), and small cell carcinomas of the lung (SCLC) were collected from online databases. Using 374 of 451 HKGs, differentially expressed genes between pairs of sample types were determined via two-sided, homoscedastic *t*-test. Principal component analysis and hierarchical clustering classified normal lung and lung cancers subtypes according to relative gene expression variations. We used uni- and multi-variate cox-regressions to identify significant predictors of overall survival in AD patients. Classifying genes were selected using a set of training samples and then validated using an independent test set. Gene Ontology was examined by PANTHER.

**Results:**

This study showed that the differential expression patterns of 242, 245, and 99 HKGs were able to distinguish normal lung from AD, SCLC, and SQCLC, respectively. From these, 70 HKGs were common across the three lung cancer subtypes. These HKGs have low expression variation compared to current lung cancer markers (e.g., EGFR, KRAS) and were involved in the most common biological processes (e.g., metabolism, stress response). In addition, the expression pattern of 106 HKGs alone was a significant classifier of AD versus SQCLC. We further highlighted that a panel of 13 HKGs was an independent predictor of overall survival and cumulative risk in AD patients.

**Discussion:**

Here we report HKG expression patterns may be an effective tool for evaluation of lung cancer states. For example, the differential expression pattern of 70 HKGs alone can separate normal lung tissue from various lung cancers while a panel of 106 HKGs was a capable class predictor of subtypes of non-small cell carcinomas. We also reported that HKGs have significantly lower variance compared to traditional cancer markers across samples, highlighting the robustness of a panel of genes over any one specific biomarker. Using RNA-seq data, we showed that the expression pattern of 13 HKGs is a significant, independent predictor of overall survival for AD patients. This reinforces the predictive power of a HKG panel across different gene expression measurement platforms. Thus, we propose the expression patterns of HKGs alone may be sufficient for the diagnosis and prognosis of individuals with lung cancer.

## Introduction

In 1965, Watson characterized housekeeping genes (HKGs) as essential genes, those “expressed in all tissues” (Watson & Levinthal, 1965). Since then, it has been further refined as genes required for the maintenance of functions essential for a cell’s existence, ubiquitously expressed across tissue type and developmental or cell cycle stage (Eisenberg & Levanon, 2013). In addition, other studies have suggested several unique genomic features of HKGs. For example, HKGs were shown to have shorter introns and exons (Vinogradov, 2004; Eisenberg & Levanon, 2013), lower conservation of promoter sequences (Lawson & Zhang, 2008), and protein products enriched in some domain families (Lehner & Fraser, 2004). However, one important notion that needs to be re-examined is the assumption that HKGs maintain constant expression levels across all cells and conditions (Yang et al., 2002; Rubie et al., 2005).

The advent of high-throughput screening technologies such as microarrays and RNA-Seq provides the ability to formulate a more concrete description of HKGs on the genomic scale. One such early, large-scale study examined the expression levels of 7,000 genes in 11 different human adult and fetal tissues, from which Warrington et al. (2000) identified 535 HKGs that were expressed in fetal development and throughout adulthood in all tissues. Subsequently, Hsiao et al. (2001) analysed the expression pattern of 7,070 genes across 19 human tissue types to identify 451 HKGs, 358 of which were common to Warrington’s list. These two studies were particularly important as they highlighted that HKGs, while constitutively expressed across tissues, did not maintain constant expression levels (Warrington et al., 2000; Hsiao et al., 2001). Rather, Hsiao et al. (2001) demonstrated that their expression patterns were sufficient to differentiate between human tissue types.

Since HKGs can distinguish one normal human tissue type from another, it is natural to ask whether these same genes may be used in discriminating between diseased tissues. Cancer is a multifactorial disease whose characteristics shift with time and space. In a study focused on breast neoplasms, it was revealed that the most frequently used traditional HKGs (e.g., GAPDH, ACTB and TUBA1A) appeared significantly altered in expression levels from one sample to the other (Janssens et al., 2004). Byun, Logothetis & Gorlov (2009) further noted that HKGs are more likely to be differentially expressed in prostate tumorigenesis, perhaps indicating their driving role in cancer development.

For this study, we focus on a subset of 374 of the 451 HKGs (originally identified by Hsiao et al., 2001) that are common to the three standard Affymetrix microarrays containing human genes: HuGene-FL (∼7000 unique sequences), HG-U95A (∼12,000 unique sequences), and HG-U133A (∼22,000 unique sequences) (Table S1). Using this set of 374 HKGs, we can extend our analysis to disease studies using different expression platforms ranging from multiple array types (HuGene-FL and HG-U95Av2) to RNA-Seq. Our goal is to test whether the expression patterns of a cluster of HKGs can serve as markers in fingerprinting human lung cancer.

## Material and Methods

Affymetrix oligonucleotide microarray data was collected from online databases, processed, and then stored in a relational database. The database for lung cancers, containing 13 normal lung samples, 89 adenocarcinomas of the lung (AD), seven small cell lung carcinoma (SCLC), and 24 squamous cell lung carcinoma (SQCLC), using the Affymetrix U95A arrays were downloaded from the Broad Institute (http://portals.broadinstitute.org/cgi-bin/cancer/publications/view/62). Additional microarray datasets using the Affymetrix U133 arrays, GDS4794 (23 SCLC, 42 Normal), GDS3627 (40 AD, 18 SQCLC), and GDS3257 (58 AD, 49 Normal) were downloaded from NCBI’s database (https://www.ncbi.nlm.nih.gov/gds/) and used for classification of lung cancers. The Affymetrix microarray expression values were computed in each study using standard analysis tools such as the Affymetrix Microarray Suite (MAS) software, RMA, or gcRMA. Then for global normalization of each microarray dataset, the average expression signal in an array was made equal to 100.

The classification of samples for survival was performed with HKG expression data from Agilent Whole Human Genome Microarrays GSE13213 (117 AD) as the training set and the RNA-Seq data of 519 AD samples as the testing set analysed using normalized RSEM values from the TCGA-LUAD study, downloaded through UCSC Xena Browser (http://xena.ucsc.edu/).

Differentially expressed genes between pairs of sample types were determined using two-sided, homoscedastic *t*-test followed by FDR (Benjamini–Hochberg) correction with Microsoft Excel. All differences in the mean log (expression levels) between samples of two groups (e.g., lung cancer versus normal lung) in the training set were determined to be statistically significant if *p* < 0.05 following FDR (Benjamini–Hochberg) correction. In every application the use of different microarray datasets as “training” and “testing” sets as well as across different gene expression platforms (i.e., microarrays and RNA-seq) serve to minimize the effects of overfitting that are typically seen when elucidating differentially expressed genes (Tinker, Boussioutas & Bowtell, 2006).

Hierarchical clustering provided unsupervised classification of normal tissue and tumours according to relative variations in gene expression patterns of the 374 HKG’s. Hierarchical clustering computations were executed with GENE-E (https://software.broadinstitute.org/GENE-E/). Although the choice of clustering algorithm is somewhat subjective (i.e., there is no “correct” cluster algorithm for all applications) (Quackenbush, 2006), the analysis parameters we utilized in this study have been shown to accurately and reproducibly distinguish between various normal tissues based on expression levels of HKGs reliably expressed in all specimens (Hsiao et al., 2001). Samples analysed using both HuGeneFL and U95Av2 microarrays were used in cluster analysis.

Principal component analysis (PCA) and corresponding 95% confidence ellipses were performed with the R package, FactoMineR (http://factominer.free.fr). Equal numbers of samples for PCA were randomly selected from each group in a given testing set.

Survival analysis was performed over 451 HKGs with the TCGA-LUAD study. Samples missing “Overall Survival”, age, gender, and status of mutational indicators of lung cancer (KRAS, EGFR, and ALK) were excluded. To identify significant predictors of overall survival we used uni- and multi-variate cox–regressions and to examine the strength of gene expression-guided risk groups we utilized Kaplan–Meier survival analyses, both of which were performed with SPSS. Risk scores were derived from the Cox proportional hazards regression model, with the median score (−0.177) separating “high-risk” and “low-risk” groups (Parker et al., 2008). Gene Ontology examinations were through PANTHER (http://pantherdb.org), which utilizes binomial distribution test and Bonferroni correction in overrepresentation analyses.

## Results

### Housekeeping genes alone are sufficient to distinguish normal tissue from lung cancers

**Figure 1:**
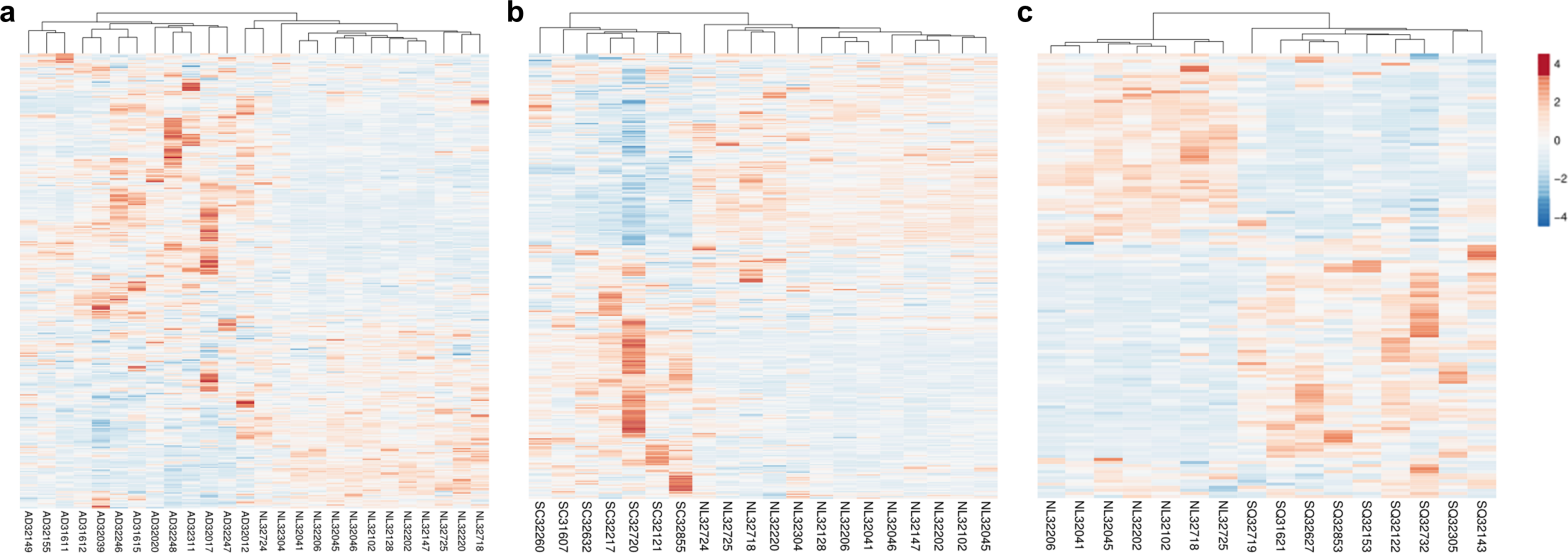
HKGs alone are sufficient to distinguish normal lung tissue from various lung cancers. Using 374 HKGs identified by previous study (7), hierarchical clustering analysis shows that (A) 242 genes identified in a training set have significant differential expression that are sufficient to separate normal lung from most lung adenocarcinoma in an independent test set; while (B) 245 genes identified in a training set can distinguish normal lung from small cell carcinoma in a test set and (C) 99 genes from a training set can distinguish normal lung from squamous cell carcinoma in a test set. *P* < 0.01 for A, B and C. NL, Normal Lung; AD, Adenocarcinoma; SC, Small Cell Carcinoma; SQ, Squamous Cell Carcinoma.

Our previous work demonstrated that the 451 HKGs are sufficient to distinguish one normal human tissue from another (Hsiao et al., 2001). In this study, we explored the possibility that 374 common HKGs can also be used to distinguish between normal and diseased tissues across different gene expression platforms and laboratories. Samples for lung cancers and normal lung tissues were used for this analysis. Expression data of 49 normal lung tissues and 58 AD from GDS3257 using the Affymetrix U133A arrays at the NIH were used as a “training set”. Two-tailed *t*-test highlighted 242 differentially expressed HKGs between the AD and normal lung tissues. These HKGs were then used to classify the microarray data from the Broad Institute using the Affymetrix U95A arrays (“testing set”). The resulting hierarchical cluster demonstrates that HKGs can separate the normal lung from AD (Fig. 1A).

The same process was repeated with the goal of identifying gene expression patterns between normal tissue, SCLC and SQCLC. Once again, training sets were utilized to select for differentially expressed genes. For SCLC analysis, 42 normal and 23 SCLC samples for the training set were from GDS4794 using the Affymetrix U133 Plus 2 arrays in Japan. In the SQCLC study, 12 SQCLC and six normal randomly selected samples from the Broad Institute were used for the training set. Distinct clustering was accomplished with 245 HKGs in 13 normal versus seven SCLC test samples from the Broad (Fig. 1B) and 99 HKGs in the remaining seven normal versus nine SQCLC test samples also from the Broad dataset (Fig. 1C; Table S2).

**Figure 2:**
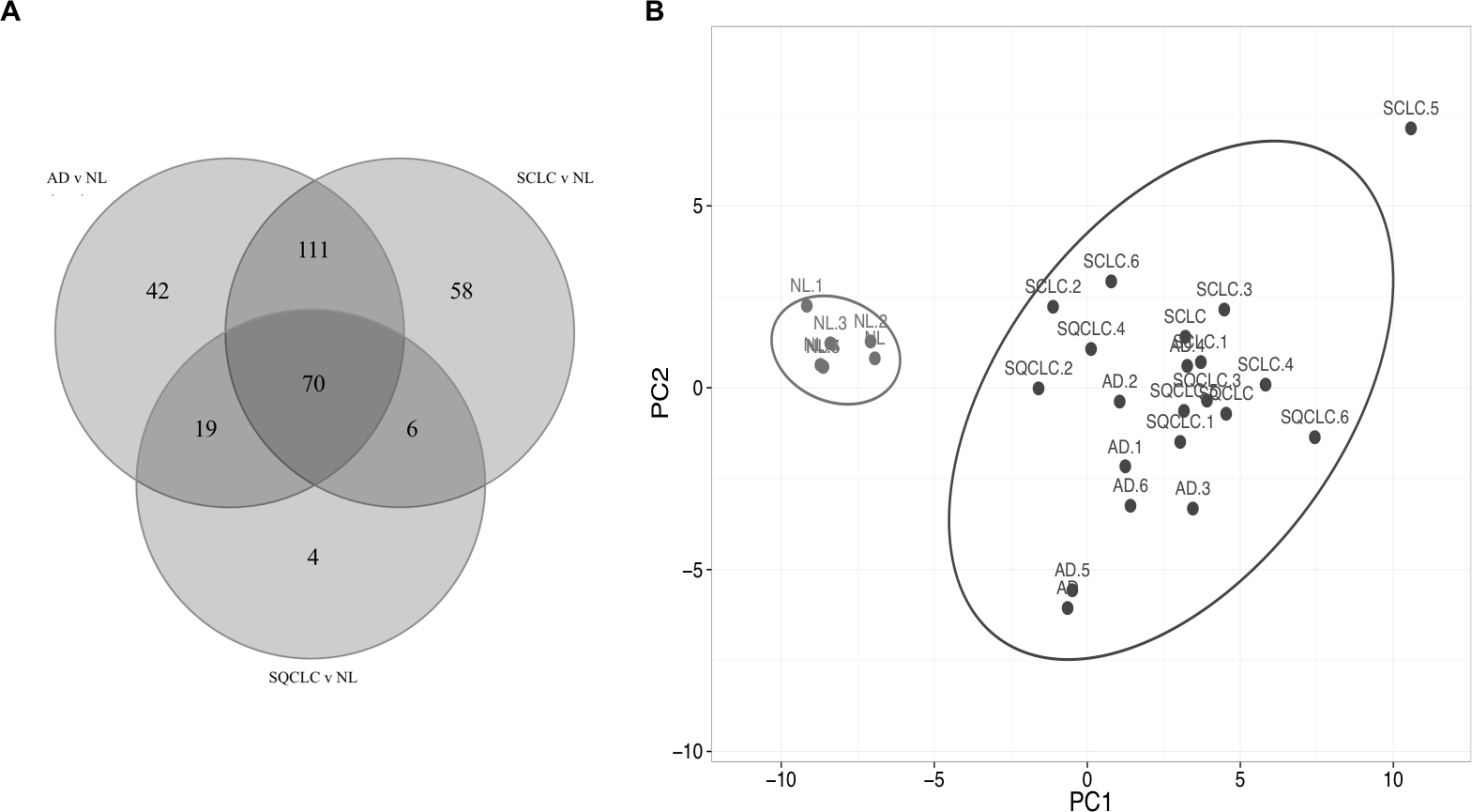
Cluster of normal lung tissue from lung cancer samples demonstrates separation power of using 70 HKGs. The numeric labels indicate the number of genes used to differentiate normal lung versus cancers shown in Fig. 1. (A) There are 70 HKGs with significant differential expression patterns common to AD, SCLC and SQCLC. (B) These 70 HKGs alone were able to achieve separation in an independent test set of six randomly selected normal lung tissues from AD, SCLC and SQCLC. NL, Normal Lung; AD, Adenocarcinoma, SCLC, Small Cell Carcinoma; SQCLC, Squamous Cell Carcinoma.

Comparison of the three gene lists used in the Fig. 1 allowed us to identify 70 differentially expressed HKGs that are common among the lung cancer samples such as 26S protease regulatory subunit 4 (PSMC1), ubiquitin-conjugating enzyme E2 C (UBE2C), ubiquitin-conjugating enzyme E2 D3 (UBE2D3), proteasomal non-catalytic subunit (PSMD2), ras homolog family member A (RHOA), and FYN proto-oncogene, Src family tyrosine kinase (FYN) (Fig. 2A, Table S3). We report here that the expression patterns of these 70 HKGs alone could separate six normal lung tissues from 18 (six AD, six SCLC, six SQCLC) cancerous tissues in the Broad test set using principal component analysis (Fig. 2B). The results were further confirmed via unsupervised hierarchical clustering and k-means clustering analysis (Figs. S1,  S2). Ontology analysis revealed that these 70 genes were largely involved in the most common biological processes such as those involved in metabolism, cell cycle regulation, and stress and immune response (Table 1). Many studies have shown that the four most represented pathways of the 70 HKGs (integrin signalling, ubiquitin proteasome, EGF receptor signalling, and FGF signalling) have been reported to regulate cancer growth and metastasis in multiple cancer types (Czubayko et al., 1997; Tamura et al., 1999; Frezza, Schmitt & Ping Dou, 2011).

**Table 1:** Ontology and pathway analysis of 70 shared genes among the three lung cancer subtypes (AD, SCLC, SQCLC).

Biological process: -Primary metabolic process -Cell communication -Cell cycle regulation -Stress response -Immune response
Molecular function: -Protein binding -Nucleic acid binding -Hydrolase activity -Enzyme regulator activity -Structural constituent of cytoskeleton
Cellular components: -Intracellular -Nucleus -Plasma membrane -Protein complex
Pathway: -Integrin signaling pathway -Ubiquitin proteasome pathway -EGF Receptor signaling pathway -FGF signaling pathway

### The 70 diagnostic housekeeping genes exhibit low variation across and within multiple lung cancer subtypes compared to current disease markers

We used six known lung cancer markers, epidermal growth factor receptor (EGFR), anaplastic lymphoma receptor tyrosine kinase (ALK), GTPase KRas (KRAS), hepatocyte growth factor receptor (MET), phosphatidylinositol 4,5-bisphosphate 3-kinase catalytic subunit alpha isoform (PIK3CA), and ret proto-oncogene (RET) to compare the coefficient of variation (CV) of our 70 HKGs set with cancer datasets. Using the Broad dataset, we showed that KRAS has the lowest average CV, 0.54, among the six selected markers across 120 lung cancer samples. In contrast, 62 out of our 70 genes had an average CV across the same samples less than 0.54 (Table S3). Examining the 120 lung cancer samples, only UBE2C and dual specificity protein phosphatase 1 (DUSP1) had average CVs greater than 1 (1.06 and 1.04, respectively). The low variance of these 70 genes across three major lung cancer types suggests a common set of genetic drivers may be involved in lung cancer development. In addition, cancer subtype analysis showed that the known cancer markers have relatively high variation within the subtypes, for example Table 2 shows that ALK had a CV of 5.19 in AD of the lung, 1.69 in SCLC and 10.33 in SQCLC. Similarly, RET had a CV of 0.44 in SCLC, 4.90 in AD and 5.10 in SQCLC. In contrast, among our 70 genes, Table S3 shows that UBE2C has the greatest CV, 1.91 in AD, markedly lower than the CVs seen in commonly known cancer marker, ALK and RET. Furthermore, Table 2 shows that the 10 HKGs with the lowest CVs are smaller than 0.34. The relatively low variance of the 70 HKGs within the normal samples and the cancer subtypes may improve their clinical reliability in serving as cancer biomarkers.

**Table 2:** Top 10 HKGs with the lowest average coefficient of variation. Among the 70 shared HKGs, these 10 genes have a CV less than 0.34 and a maximum difference in CV of 0.167 (in HYAL2) across cancer subtypes.

			Coefficient of variation			
Accession number	Gene name	Official symbol	NL	AD	SCLC	SQCLC
P61077	Ubiquitin-conjugating enzyme E2 D3	**UBE2D3**	0.204	0.204	0.142	0.257
P61586	Transforming protein RhoA	**RHOA**	0.087	0.179	0.204	0.223
P27348	14-3-3 protein theta	**YWHAQ**	0.111	0.241	0.177	0.213
Q16531	DNA damage-binding protein 1	**DDB1**	0.074	0.199	0.165	0.296
Q13418	Integrin-linked protein kinase	**ILK**	0.191	0.229	0.260	0.193
P07910	Heterogeneous nuclear ribonucleoproteins C1/C2	**HNRNPC**	0.124	0.189	0.307	0.188
Q12891	Hyaluronidase-2	**HYAL2**	0.266	0.194	0.339	0.172
P55061	Bax inhibitor 1	**TMBIM6**	0.168	0.278	0.257	0.180
P50991	T-complex protein 1 subunit delta	**CCT4**	0.126	0.293	0.128	0.298
Q15233	Non-POU domain-containing octamer-binding protein	**NONO**	0.081	0.250	0.234	0.238
P01116	Kirsten rat sarcoma viral oncogene homolog	KRAS	0.240	1.027	0.330	0.262
Q9UM73	ALK receptor tyrosine kinase	ALK	2.066	5.190	1.692	10.334
P07949	ret proto-oncogene	RET	1.842	4.899	0.440	5.100
P00533	Epidermal growth factor receptor	EGFR	0.413	1.897	1.408	0.903
P08581	Hepatocyte growth factor receptor	MET	0.961	1.918	1.413	0.836
P42336	Phosphatidylinositol 4,5-bisphosphate 3-kinase catalytic subunit alpha isoform	PIK3CA	0.367	0.561	0.655	0.666

**Notes.**

a Bolded gene symbols are housekeeping genes.

TITLE NL Normal Lung AD Adenocarcinoma SCLC Small Cell Lung Carcinoma SQCLC Squamous Cell Lung Carcinoma

### Housekeeping genes alone can also differentiate lung adenocarcinoma from squamous cell carcinoma

**Figure 3:**
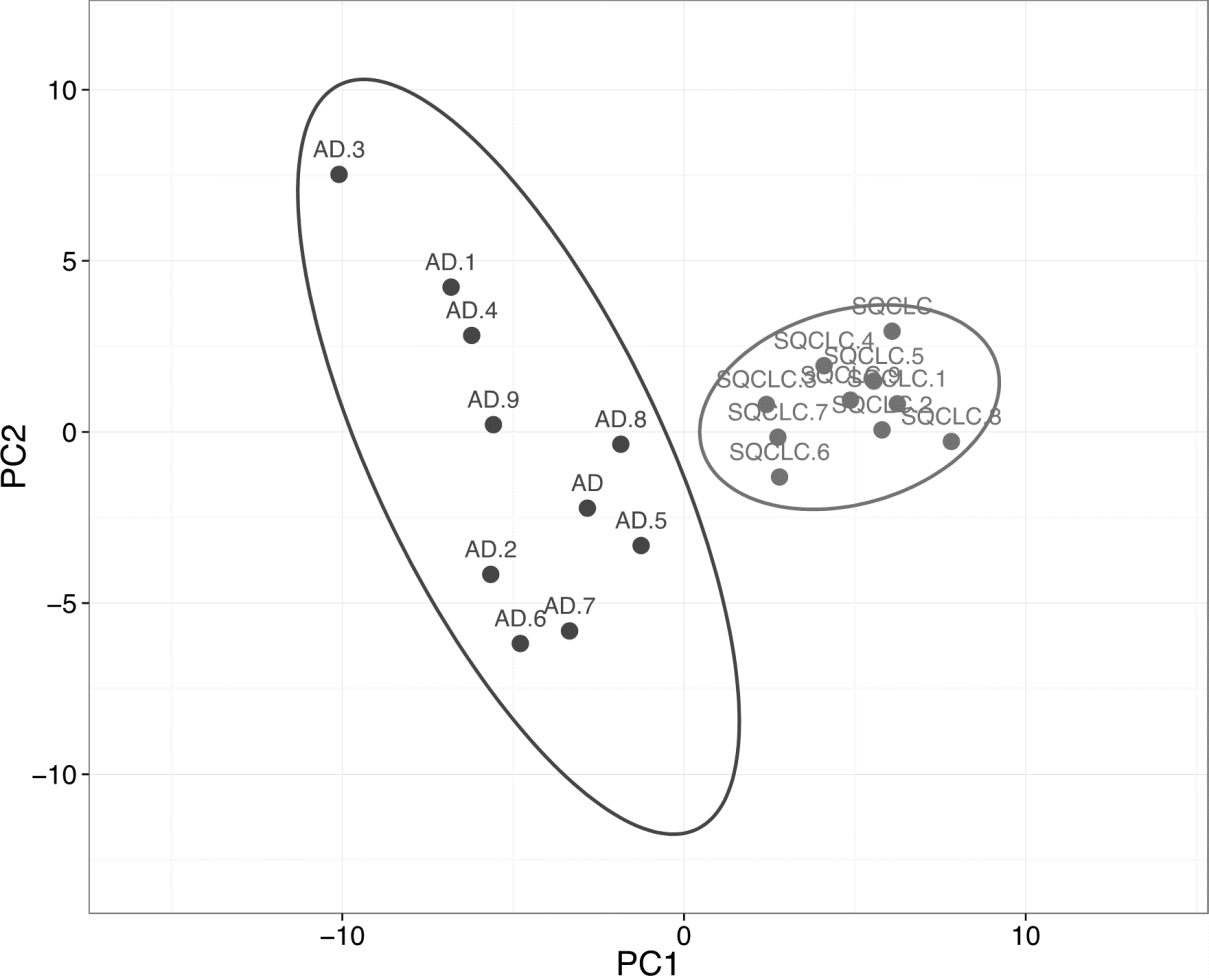
Expression of 106 HKGs is a significant class predictor for subsets of non-small cell lung cancers. Ten samples each of AD and SQCLC were randomly selected as testing set, which were able to be distinguished by 106 differentially expressed HKGs. AD, Adenocarcinoma; SQCLC, Squamous Cell Carcinoma.

We then assessed the ability of HKGs to distinguish two subtypes of non-small cell carcinoma (NSCLC). Utilizing GDS3627 data, with 40 AD and 18 SQCLC selected as the training set, we identified 106 differentially expressed genes between AD and SQCLC (Table S4). Additional assessment with PCA showed that the expression patterns of these 106 genes displayed clear separation of AD and SQCLC in a test set comprised of 10 randomly selected samples for AD and SQCLC from the Broad dataset. This finding reinforces the value of HKGs as class predictors (Fig. 3).

While neurofilament light polypeptide (NEFL) and dishevelled segment polarity protein 3 (DVL3) show fold changes of 3.26 and 2.09, respectively, the remaining 104 HKGs have fold changes less than 2. A total of 34 different pathways were associated with the 106 HKGs. Examination of 15 pathways containing the greatest number of the 106 HKGs reveals those critical in cancer development such as apoptosis signalling, angiogenesis, p53, and glycolysis (Table 3). Furthermore, many genes are involved in multiple different pathways. For example, A-Raf proto-oncogene, serine/threonine kinase (ARAF) appears in the angiogenesis pathway as well as integrin, VEGF, FGF, and EGF signalling. In addition, ARAF comprises known functions involving cancer development such as activation of MAPK/ERK (associated with cell growth) and binding to pyruvate kinase isozymes M1/M2 (PKM2), critical for the Warburg effect. Furthermore, the 106 HKGs were also reported to link to diseases such as Alzheimer, Parkinson, and Huntington. Interestingly, the differentially expressed HKGs associated with the ubiquitin proteasome pathway all had lower expression in AD compared to SQCLC. The wide range of diseases and processes associated with the 106 HKGs highlights their roles in basic cellular processes and maintenances.

**Table 3:** Examples of pathways involved in 106 differentially expressed genes between AD and SQCLC.

Pathway	Genes
Huntington disease	**NCOR2**, **GAPDH**, **ARF4**, **CAPN2**, ARF3, TUBB, DYNLL1
Angiogenesis	**ARHGAP1**, **ARAF,** DVL3, HSPB1
Ubiquitin proteasome pathway	PSMC1, UBE2C, PSMD2, PSMD7
Alzheimer disease	**NCSTN**, DVL3, PSEN1
Integrin signaling pathway	**ARAF**, **ILK**, ARF3
VEGF signaling pathway	**ARHGAP1**, **ARAF**, HSPB1
FGF signaling pathway	**ARAF**, YWHAZ, YWHAQ
EGF receptor signaling pathway	**ARAF**, YWHAZ, YWHAQ
Parkinson disease	**SEPT2**, YWHAZ, YWHAQ
Cytoskeletal regulation by Rho GTPase	**ARHGAP1**, TUBB, STMN1
Notch signaling pathway	**NCOR2**, **NCSTN**, PSEN1
Apoptosis signaling pathway	**TMBIM6**, ATF4
p53 pathway	HDAC1, HMGB1
Glycolysis	**GAPDH**, PGAM1
Wnt signaling pathway	HDAC1, DVL3

**Notes.**

a Bolded gene symbols have higher expression in AD compared to SQCLC.

### Housekeeping gene expression is an independent predictor of overall survival in lung Adenocarcinoma

Much of the focus on individual gene expression has been on its value as a prognostic marker in disease states (Beer et al., 2002; Shedden et al., 2008). Here we demonstrate that a panel of HKGs may be a valuable tool in predicting overall survival for AD. The list of HKGs was first ranked by log-rank test *P*-values gained through Cox-regression analysis of the training set ( GSE13213) data for 116 patients using Agilent arrays. Survival times for the 116 patients are listed in Table S5. Utilizing the resulting list of 13 significant HKGs (*p* < 0.05, unadjusted) from the training set (Table 4), samples from the testing set (TCGA-LUAD, RNA-Seq data for 576 patients) were plotted on a continuous risk curve; the median score (−0.177) separated “low” and “high” risk groups. These risk groups, classified by expression of 13 HKGs, showed average difference of 2,078 days in overall survival (mean low risk-mean high risk) (Table 5). To test whether this response was a viable and independent predictor, we performed multivariate analyses to include age, gender, and the presence of known mutational drivers (KRAS, EGFR, and ALK) (Table 6). Our risk group classification was the only significant estimator of overall survival.

**Table 4:** Thirteen HKGs significant in predicting overall survival in patients with AD.

Accession number	Gene name	Official symbol	*P*-value (training)	*P*-value (testing)	Fold change (testing)
P21333	Filamin-A	FLNA	0.000	0.140	2.00
P62873	Guanine nucleotide-binding protein subunit beta-1	GNB1	0.033	0.465	2.01
P50914	60S ribosomal protein L14	RPL14	0.004	0.513	2.03
P62899	60S ribosomal protein L31	RPL31	0.003	0.918	2.03
Q06323	Proteasome activator complex subunit 1	PSME1	0.032	0.359	2.04
O00483	Cytochrome c oxidase subunit NDUFA4	NDUFA4	0.012	0.378	2.04
P61343	60S ribosomal protein L27	RPL27	0.024	0.090	2.07
A5PKW4	Pleckstrin and Sec7 domain containing	PSD	0.016	0.000	0.43
P32969	60S ribosomal protein L9	RPL9	0.038	0.038	0.49
Q14011	Cold-inducible RNA-binding protein	CIRBP	0.011	0.031	0.49
P20807	Calpain-3	NCL	0.015	0.094	0.50
P30084	Enoyl-CoA hydratase, mitochondrial	ECHS1	0.037	0.221	0.50
P18124	60S ribosomal protein L7	RPL7	0.014	0.793	0.50

**Table 5:** Mean and median survival times for two risk groups of patients with adenocarcinoma of lung using 13 HKGs.

	Mean				Median			
			95% confidence				95% confidence	
	Survival (days)	Std. error	Lower	Upper	Survival (days)	Std. error	Lower	Upper
Low risk group	3,560.98	352.23	2,870.61	4,251.35	2,617	384.62	1,863.14	3,370.85
High risk group	1,482.93	213.60	1,064.27	1,901.58	995	80.80	836.64	1,153.35
Difference	2,078.35				1,619			

**Table 6:** Multivariable hazards analysis of overall survival for patients with lung adenocarcinoma using 13 HKGs. Compared to the presence of KRAS/EGFR/ALK mutations, age, and gender of patients, risk group classification by the expression of 13 HKGs is the only significant predictor of overall survival using multivariable hazards analysis.

			95% confidence	
	*P*-value	Hazard ratio	Lower	Upper
Risk group	0	3.29	2.20	4.92
KRAS/EGFR/ALK mutation	0.268	0.98	0.66	1.45
Age	0.508	1.01	0.99	1.03
Gender	0.052	0.70	0.48	1.03

**Figure 4:**
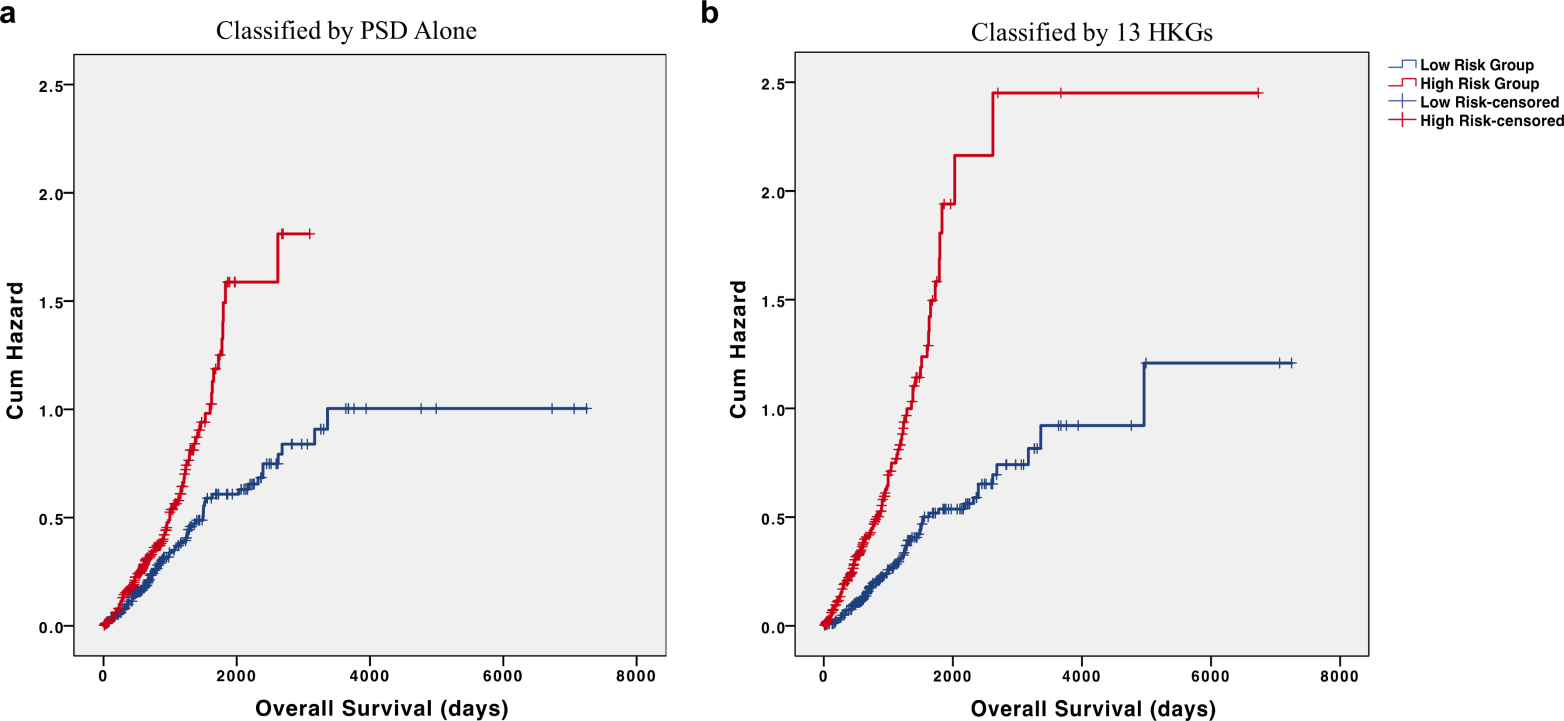
HKGs are significant predictors of long-term hazard in patients with adenocarcinoma. Classification of individuals with AD into “high” or “low” risk groups by (A) PSD expression alone led to a marked decrease in the difference of cumulative hazard of the two risk groups when compared to classification by (B) 13 HKGs expression.

Interestingly, most of the 13 individual HKGs were not individually significant predictors of overall survival in the testing set. Indeed, the greatest differences were an average 2.32-fold decrease in pleckstrin and Sec7 domain containing (PSD) and 2.07-fold increase in 60S ribosomal protein L27 (RPL27) in the high-risk group (Table 4). In the testing set, all but three of these HKGs were not individually significant (*p* > 0.05); however, their removal from the panel led to a marked decrease in predictive value. As PSD was determined as the most significant individual gene in our testing set and had one of the highest fold changes, we utilized PSD alone to determine risk groups. Our results show a marked reduction in differences of cumulative hazard between the two groups from classification by 13 HKGs (Fig. 4). This finding reinforces the value of a panel of genes over any one specific biomarker in disease prognosis.

While 5 out of the 13 HKGs are ribosomal protein-related genes, not all the ribosomal protein encoding genes responded in the same way. RPL14, RPL31, and RPL27 all displayed increases in high-risk individuals whereas RPL9 and RPL7 were decreased. Despite studies having examined the roles of RPLs in the regulation of the MDM2/MDMX–p53 cascade, much remains unknown on how individual RPLs may function in cancer progression (Zhang et al., 2003; Zhou et al., 2015).

## Discussion

Much of the efforts studying disease states using microarray and RNA-Seq technology have focused on the identification of “specific disease markers” (Bubendorf et al., 1999; Golub et al., 1999; Glynne et al., 2000). Although the value of these efforts is undeniable, it remains a challenge to find suitable analytical tools that will provide reliable conclusions. As we have discussed previously, the gene expression patterns in any given sample represents the specific cellular states of the sample at the time of collection (Hsiao et al., 2001). In other words, cancers at different stages may express different markers. For example, the use of prostate-specific antigen in testing for prostate cancer has yielded much criticism due to its high false positive rates (Potts, 2000). Similarly, while BRCA1 does significantly increase risk of breast cancer, germline mutations of this gene only account for 3% of all breast cancer cases (Whittemore, Gong & Itnyre, 1997). Therefore, there have been efforts towards examining the relationship between sets of genes or utilizing a panel of biomarkers. For example, our previous study highlights that using 3 sets of ratios, instead of one set, between two genes increased differential diagnosis accuracy from 90% to 99% in lung cancer and mesothelioma (Gordon et al., 2002).

HKGs offer a potential solution to the problems of specific markers. Their constitutive, and relatively high, expressions in all cells provide assurance of being identifiable in any sample (Caracausi et al., 2017). Furthermore, studies have shown that HKGs cannot be assumed to maintain constant expression levels in all cells and conditions (Hsiao et al., 2001; Barber et al., 2005). Others have also examined the states of HKGs in cancers; Blanquicett et al. notes that 15 traditional HKGs (e.g., PGK, GAPDH, and *β*-Actin) demonstrate significant expression variations between carcinomatous and normal liver samples (Blanquicett et al., 2002). Similarly, Rubie et al. found that HKGs that code for the metabolic enzymes, PGK and GAPDH, show high up-regulation in cancerous versus normal tissues from the pancreas, stomach, and colon (Rubie et al., 2005). In our study, we show that the expression patterns of 70 HKGs alone can significantly differentiate between normal and lung cancer samples.

Our study also highlights that these 70 HKGs maintain low levels of variance across tumour samples when compared to accepted markers of lung cancer (e.g., KRAS, ALK, RET). As HKGs are largely responsible for basic cellular maintenance, this suggests that our identified list of HKGs may be the common drivers of lung cancer development. While some of the 70 HKGs (e.g., PSMD2) have been identified by other genome-wide association studies as relevant in single subtypes of lung cancer development (AD), many have not been fully examined, adding to the list of possible targets for therapeutic development (Matsuyama et al., 2011). In addition, other HKGs have been seen to drive pathogenesis across tissue types. For example, overexpression of ubiquitin-conjugating enzyme E2C (UBE2C) has recently been thought to play a role in not only NSCLC, but also gastric, colorectal, and breast cancers (Wang et al., 2015; Pellino et al., 2016; Yang et al., 2016). Despite this low variance of HKGs across lung cancer samples, our study shows that a panel of only 106 differentially expressed HKGs was able to act as a class predictor between two subtypes of NSCLC (SQCLC and AD).

Furthermore, the expression patterns of 13 HKGs alone provided significant prognostic value, estimating 2,078 days in overall survival difference between the high and low risk groups. These results are independent of traditional clinical markers. Importantly, we utilized separate datasets for training and testing to circumvent issues of overfitting. Moreover, our training set being microarray data and our testing set being from RNA-Seq demonstrates the robustness of a panel of 13 HKGs across different gene expression measurement technologies.

The results of this study are consistent with the idea that while HKGs may maintain relatively stable gene expressions among similar tissue-type samples, their expression profiles remain tissue and disease specific (Warrington et al., 2000; Hsiao et al., 2001). The disease-specificity of HKG expressions demonstrates the predictive capabilities for lung cancers, while its ubiquitous and low intra-tissue variance allows for its reproducibility across different microarray platforms and RNA-seq technology. However, there remains translational challenges. For example, it has been shown that different microarray platforms displayed varying degrees of repeatability, reproducibility, and consistency (Consortium, 2006; Shi et al., 2008). Due to lack of public datasets, this study was largely constrained to two microarray types (HuGeneFL and U95v2).

## Conclusions

Overall, our findings reinforce both the diagnostic and prognostic power of HKG expression patterns. Given their ubiquitous nature and role in maintenance of basic cellular functions, it is possible that HKGs act as drivers in lung cancer. Thus, while it has been reported that HKGs evolve at a slower rate than other genes (Zhang & Li, 2004), it will be of interest to examine the mutational patterns of HKGs and further validate their relevance in lung cancer development. Furthermore, as we have demonstrated, HKGs is a strong predictor of “low” and “high” risk patients whom differ significantly in overall survival. It is therefore important that future studies examine the correlation of HKG patterns to the morphology and histology of lung cancer tissues; because while histology may provide initial stratification of patients into rough classes, HKGs may offer a more reliable method of classification. Consequently, the ability to identify high-risk individuals in early lung cancer stages may allow for adjustments of therapeutic interventions and increased survival.

##  Supplemental Information

10.7717/peerj.4719/supp-1 Figure S1 Normal lung samples are distinctly separated from cancerous lung samples by 70 HKGs Utilizing the 70 HKGs All samples from the Broad Institute Affymetrix U95A arrays were subjected to unsupervised hierarchical clustering analysis using average distance.

10.7717/peerj.4719/supp-2 Figure S2 Confirmation of 70 HKGs Clustering Power by separation of normal lung samples from cancerous lung samples with K-means clustering (A) 7 normal lung samples and 7 samples each of lung adenocarcinomas, squamous cell carcinomas of the lung, and small cell carcinomas of the lung were subjected to PCA by 70 HKGs. (B) The pattern seen in PCA is confirmed by K-means clustering (Number of Clusters = 2, Iterations = 50, Attempts = 50); Furthermore, when the number of clusters was increased to 5 (C) the separation did not improve. NL, Normal Lung; AD, Adenocarcinoma; SC, Small Cell Carcinoma; SQ, Squamous Cell Carcinoma.

10.7717/peerj.4719/supp-3 Table S1 374 Housekeeping Genes common to three standard Affymetrix microarrays

10.7717/peerj.4719/supp-4 Table S2 List of Squamous Cell Lung Carcinoma (SQ) and Normal Lung (NL) Samples from the Broad Institute Dataset in either Training or Testing Sets

10.7717/peerj.4719/supp-5 Table S3 Coefficient of Variation for 70 Housekeeping Genes differentially expressed across multiple lung cancer subtypes

10.7717/peerj.4719/supp-6 Table S4 106 Housekeeping Genes differentially expressed between lung adenocarcinoma and lung squamous cell carcinomas

10.7717/peerj.4719/supp-7 Table S5 Survival times for 116 patients from GSE13213

## Additional Information and Declarations

### Data Availability

The following information was supplied regarding data availability: Raw data was gathered from online databases and details are provided in the article.

## References

[ref-1] Barber RD, Harmer DW, Coleman RA, Clark BJ (2005). GAPDH as a housekeeping gene: analysis of GAPDH mRNA expression in a panel of 72 human tissues. Physiological Genomics.

[ref-2] Beer DG, Kardia SLR, Huang C-C, Giordano TJ, Levin AM, Misek DE, Lin L, Chen G, Gharib TG, Thomas DG, Lizyness ML, Kuick R, Hayasaka S, Taylor JMG, Iannettoni MD, Orringer MB, Hanash S (2002). Gene-expression profiles predict survival of patients with lung adenocarcinoma. Nature Medicine.

[ref-3] Blanquicett C, Johnson MR, Heslin M, Diasio RB (2002). Housekeeping gene variability in normal and carcinomatous colorectal and liver tissues: applications in pharmacogenomic gene expression studies. Analytical Biochemistry.

[ref-4] Bubendorf L, Kononen J, Koivisto P, Schraml P, Moch H, Gasser TC, Willi N, Mihatsch MJ, Sauter G, Kallioniemi OP (1999). Survey of gene amplifications during prostate cancer progression by high-throughout fluorescence *in situ* hybridization on tissue microarrays. Cancer Research.

[ref-5] Byun J, Logothetis CJ, Gorlov IP (2009). Housekeeping genes in prostate tumorigenesis. International Journal of Cancer.

[ref-6] Caracausi M, Piovesan A, Antonaros F, Strippoli P, Vitale L, Pelleri MC (2017). Systematic identification of human housekeeping genes possibly useful as references in gene expression studies. Molecular Medicine Reports.

[ref-7] Consortium M (2006). The MicroArray Quality Control (MAQC) project shows inter- and intraplatform reproducibility of gene expression measurements. Nature Biotechnology.

[ref-8] Czubayko F, Liaudet-Coopman EDE, Aigner A, Tuveson AT, Berchem GJ, Wellstein A (1997). A secreted FGF-binding protein can serve as the angiogenic switch in human cancer. Nature Medicine.

[ref-9] Eisenberg E, Levanon EY (2013). Human housekeeping genes, revisited. Trends in Genetics.

[ref-10] Frezza M, Schmitt S, Ping Dou Q (2011). Targeting the ubiquitin-proteasome pathway: an emerging concept in cancer therapy. Current Topics in Medicinal Chemistry.

[ref-11] Glynne R, Ghandour G, Rayner J, Mack DH, Goodnow CC (2000). B-lymphocyte quiescence, tolerance and activation as viewed by global gene expression profiling on microarrays. Immunological Reviews.

[ref-12] Golub TR, Slonim DK, Tamayo P, Huard C, Gaasenbeek M, Mesirov JP, Coller H, Loh ML, Downing JR, Caligiuri MA, Bloomfield CD, Lander ES (1999). Molecular classification of cancer: class discovery and class prediction by gene expression monitoring. Science.

[ref-13] Gordon GJ, Jensen RV, Hsiao L-L, Gullans SR, Blumenstock JE, Ramaswamy S, Richards WG, Sugarbaker DJ, Bueno R (2002). Translation of microarray data into clinically relevant cancer diagnostic tests using gene expression ratios in lung cancer and mesothelioma. Cancer Research.

[ref-14] Hsiao L-L, Dangond F, Yoshida T, Hong R, Jensen RV, Misra J, Dillon W, Lee KF, Clark KE, Haverty P, Weng Z, Mutter GL, Frosch MP, MacDonald ME, Milford EL, Crum CP, Bueno R, Pratt RE, Mahadevappa M, Warrington JA, Stephanopoulos G, Stephanopoulos G, Gullans SR (2001). A compendium of gene expression in normal human tissues. Physiological Genomics.

[ref-15] Janssens N, Janicot M, Perera T, Bakker A (2004). Housekeeping genes as internal standards in cancer research. Molecular Diagnosis.

[ref-16] Lawson MJ, Zhang L (2008). Housekeeping and tissue-specific genes differ in simple sequence repeats in the 5′-UTR region. Gene.

[ref-17] Lehner B, Fraser AG (2004). Protein domains enriched in mammalian tissue-specific or widely expressed genes. Trends in Genetics.

[ref-18] Matsuyama Y, Suzuki M, Arima C, Huang QM, Tomida S, Takeuchi T, Sugiyama R, Itoh Y, Yatabe Y, Goto H, Takahashi T (2011). Proteasomal non-catalytic subunit PSMD2 as a potential therapeutic target in association with various clinicopathologic features in lung adenocarcinomas. Molecular Carcinogenesis.

[ref-19] Parker J, Mullins M, Cheang MC, Davies S, Mardis E, Nielsen TO, Ellis MJ, Marron S, Perou CM, Bernard PS (2008). A supervised risk predictor of breast cancer based on biological subtypes. Journal of Clinical Oncology.

[ref-20] Pellino G, Pallante P, Malapelle U, Ferraro A, Bellevicine C, Milone M, Troncone G, Fusco A, Selvaggi F (2016). UbcH10 overexpression is less pronounced in older colorectal cancer patients. International Journal of Colorectal Disease.

[ref-21] Potts JM (2000). Prospective identification of National Institutes of Health category IV prostatitis in men with elevated prostate specific antigen. Journal of Urology.

[ref-22] Quackenbush J (2006). Microarray analysis and tumor classification. New England Journal of Medicine.

[ref-23] Rubie C, Kempf K, Hans J, Su T, Tilton B, Georg T, Brittner B, Ludwig B, Schilling M (2005). Housekeeping gene variability in normal and cancerous colorectal, pancreatic, esophageal, gastric and hepatic tissues. Molecular and Cellular Probes.

[ref-24] Shedden K, Taylor JMG, Enkemann SA, Tsao MS, Yeatman TJ, Gerald WL, Eschrich S, Jurisica I, Giordano TJ, Misek DE, Chang AC, Zhu CQ, Strumpf D, Hanash S, Shepherd FA, Ding K, Seymour L, Naoki K, Pennell N, Weir B, Verhaak R, Ladd-Acosta C, Golub T, Gruidl M, Sharma A, Szoke J, Zakowski M, Rusch V, Kris M, Viale A, Motoi N, Travis W, Conley B, Seshan VE, Meyerson M, Kuick R, Dobbin KK, Lively T, Jacobson JW, Beer DG (2008). Gene expression-based survival prediction in lung adenocarcinoma: a multi-site, blinded validation study. Nature Medicine.

[ref-25] Shi L, Jones WD, Jensen RV, Harris SC, Perkins RG, Goodsaid FM, Guo L, Croner LJ, Boysen C, Fang H, Qian F, Amur S, Bao W, Barbacioru CC, Bertholet V, Cao X, Chu T-M, Collins PJ, Fan X, Frueh FW, Fuscoe JC, Guo X, Han J, Herman D, Hong H, Kawasaki ES, Li Q-Z, Luo Y, Ma Y, Mei N, Peterson RL, Puri RK, Shippy R, Su Z, Sun Y, Sun H, Thorn B, Turpaz Y, Wang C, Wang S, Warrington JA, Willey JC, Wu J, Xie Q, Zhang L, Zhang L, Zhong S, Wolfinger RD, Tong W (2008). The balance of reproducibility, sensitivity, and specificity of lists of differentially expressed genes in microarray studies. BMC Bioinformatics.

[ref-26] Tamura M, Gu J, Tran H, Yamada KM (1999). PTEN gene and integrin signaling in cancer. Journal of the National Cancer Institute.

[ref-27] Tinker AV, Boussioutas A, Bowtell DDL (2006). The challenges of gene expression microarrays for the study of human cancer. Cancer Cell.

[ref-28] Vinogradov AE (2004). Compactness of human housekeeping genes: selection for economy or genomic design?. Trends in Genetics.

[ref-29] Wang C, Pan YH, Shan M, Xu M, Bao JL, Zhao LM (2015). Knockdown of UbcH10 enhances the chemosensitivity of dual drug resistant breast cancer cells to epirubicin and docetaxel. International Journal of Molecular Sciences.

[ref-30] Warrington JA, Nair A, Mahadevappa M, Tsyganskaya M (2000). Comparison of human adult and fetal expression and identification of 535 housekeeping / maintenance genes Comparison of human adult and fetal expression and identification of 535 housekeeping/maintenance genes. Physiological Genomics.

[ref-31] Watson JD, Levinthal C (1965). Molecular biology of the gene.

[ref-32] Whittemore AS, Gong G, Itnyre J (1997). Prevalence and contribution of BRCA1 mutations in breast cancer and ovarian cancer: results from three US population-based case-control studies of ovarian cancer. American Journal of Human Genetics.

[ref-33] Yang M, Qu Y, Shi R, Wu X, Su C, Hu Z, Chang Q, Liu S, Pan G, Lei M, Xie F, Tu S, Tao W, Zhou H, Hu G, Zhang Z (2016). Ubiquitin-conjugating enzyme UbcH10 promotes gastric cancer growth and is a potential biomarker for gastric cancer. Oncology Reports.

[ref-34] Yang YH, Dudoit S, Luu P, Lin DM, Peng V, Ngai J, Speed TP (2002). Normalization for cDNA microarray data: a robust composite method addressing single and multiple slide systematic variation. Nucleic Acids Research.

[ref-35] Zhang L, Li W-H (2004). Mammalian housekeeping genes evolve more slowly than tissue-specific genes. Molecular Biology and Evolution.

[ref-36] Zhang Y, Wolf GW, Bhat K, Jin A, Allio T, Burkhart WA, Xiong Y (2003). Ribosomal protein L11 negatively regulates oncoprotein MDM2 and mediates a p53-dependent ribosomal-stress checkpoint pathway. Molecular and Cellular Biology.

[ref-37] Zhou X, Liao WJ, Liao JM, Liao P, Lu H (2015). Ribosomal proteins: functions beyond the ribosome. Journal of Molecular Cell Biology.

