# Supplementary figures and images for: Differential expression patterns of housekeeping genes increase diagnostic and prognostic value in lung cancer
Source: PeerJ. 2018 May 9;6:e4719. doi: 10.7717/peerj.4719 (PMC5949062; doi:10.7717/peerj.4719)

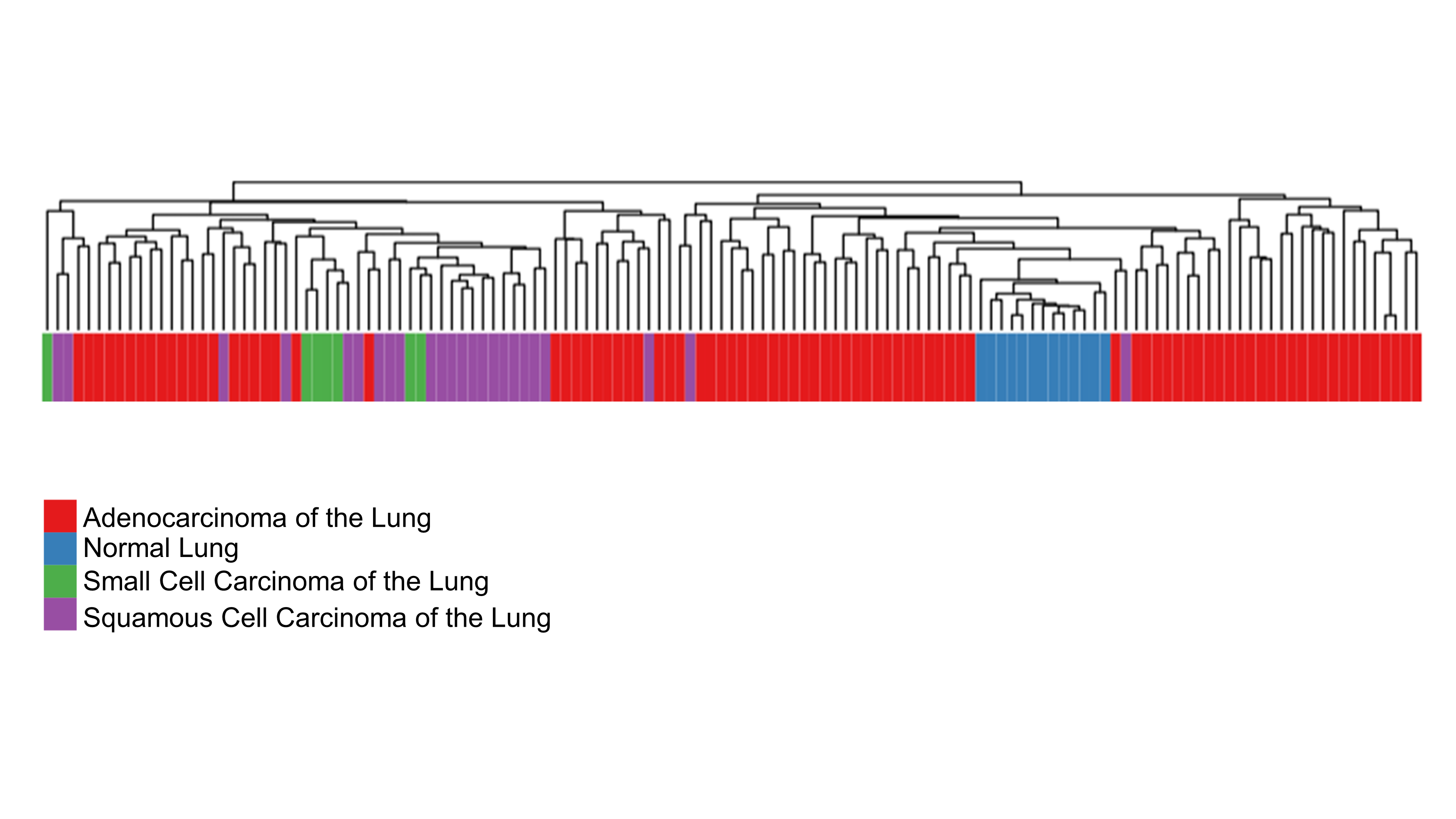

Supplement: Figure S1 — Utilizing the 70 HKGs All samples from the Broad Institute Affymetrix U95A arrays were subjected to unsupervised hierarchical clustering analysis using average distance. [file peerj-06-4719-s001.png]

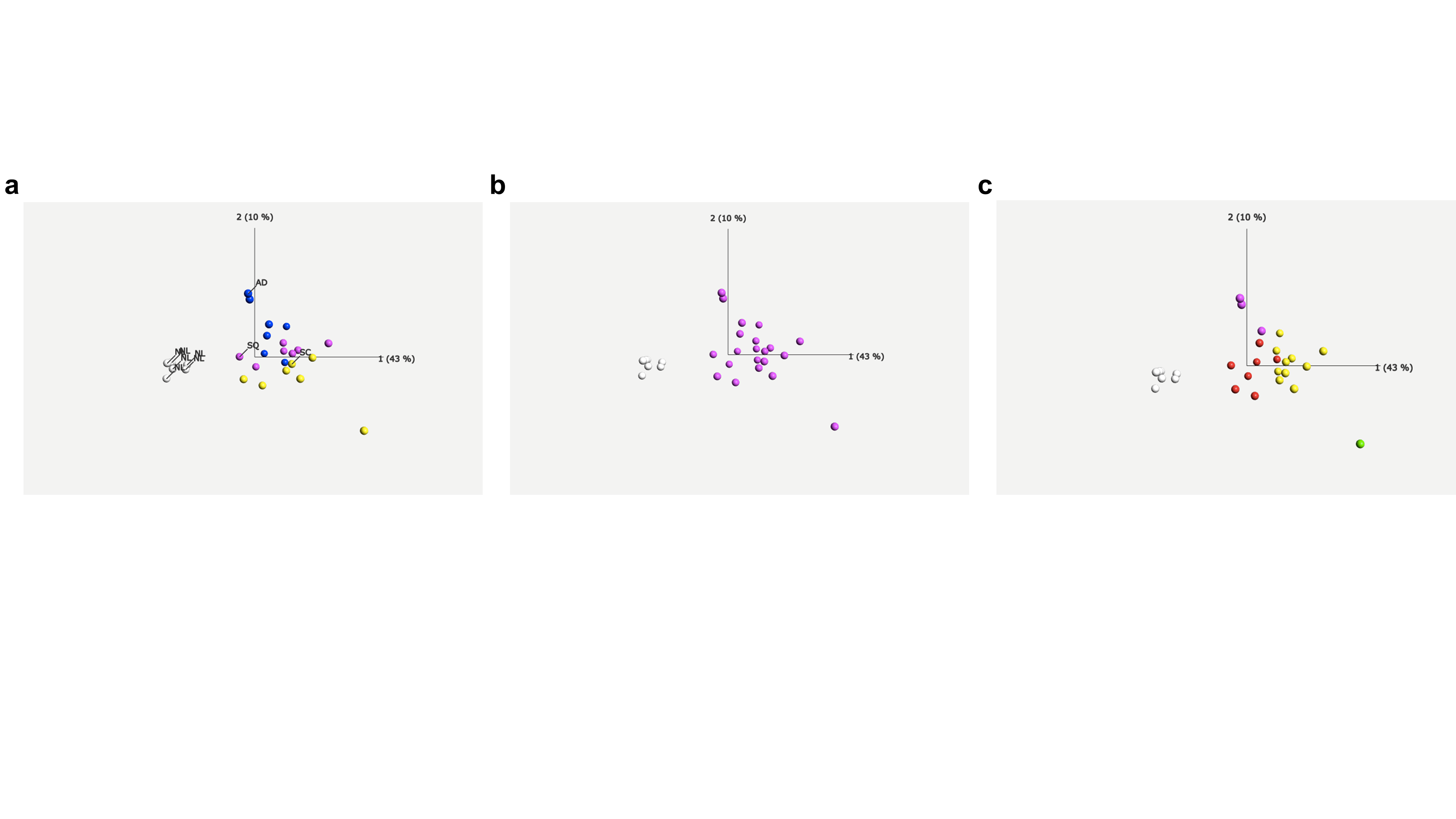

Supplement: Figure S2 — (A) 7 normal lung samples and 7 samples each of lung adenocarcinomas, squamous cell carcinomas of the lung, and small cell carcinomas of the lung were subjected to PCA by 70 HKGs. (B) The pattern seen in PCA is confirmed by K-means clustering (Number of Clusters = 2, Iterations = 50, Attempts = 50); Furthermore, when the number of clusters was increased to 5 (C) the separation did not improve. NL, Normal Lung; AD, Adenocarcinoma; SC, Small Cell Carcinoma; SQ, Squamous Cell Carcinoma. [file peerj-06-4719-s002.png]
